# Perception and Experience of Health Extension Workers on Facilitators and Barriers to Maternal and Newborn Health Service Utilization in Ethiopia: A Qualitative Study

**DOI:** 10.3390/ijerph181910467

**Published:** 2021-10-05

**Authors:** Alemayehu Hunduma Higi, Gurmesa Tura Debelew, Lelisa Sena Dadi

**Affiliations:** 1 Department of Population and Family Health, Institute of Health, Jimma University, Jimma P.O. Box 378, Ethiopia; gurmesatura@gmail.com; 2 Department of Epidemiology, Institute of Health, Jimma University, Jimma P.O. Box 937, Ethiopia; lelisa.sena@ju.edu.et

**Keywords:** perception, experience, barrier, facilitator, health extension program, health extension workers

## Abstract

*Background*: Health extension workers (HEWs) have substantial inputs to reduce maternal and newborn morbidity and mortality in Ethiopia. However, their perceptions and experiences were not well understood. Therefore, this study aimed to explore their perceptions and experiences on facilitators and barriers to maternal and newborn health services in Ethiopia. *Methods*: A descriptive qualitative study was conducted from 8–28 April 2021 in Oromia, Amhara and Southern Nation, Nationality, and People’s Regional State of Ethiopia. Focused group discussions were made with purposively selected 60 HEWs. The data were transcribed verbatim and translated into English. An inductive thematic analysis was carried out using Atlas ti.7.1. The findings were presented in major themes, categories, and sub-categories with supporting quote(s). *Results*: The findings were categorized into two major themes (i.e., facilitators and barriers) and seven sub-themes. Community-related facilitators encompass awareness and behavior at the individual, family, and community. Significant others such as traditional birth attendants, religious leaders, women developmental armies, and kebele chairman substantially contributed to service utilization. Availability/access to infrastructures such as telephone, transportation services, and solar energy systems facilitated the service utilization. Furthermore, health facility-related facilitators include the availability of HEWs; free services; supervision and monitoring; maternity waiting rooms; and access to ambulance services. Maternal and newborn health services were affected by community-related barriers (i.e., distance, topography, religious and socio-cultural beliefs/practices, unpleasant rumors, etc.,), health facility-related barriers (i.e., health worker’s behaviors; lack of logistics; lack of adequate ambulance service, and placement and quality of health post), and infrastructure (i.e., lack or poor quality of road and lack of water). *Conclusions*: The HEWs perceived and experienced a wide range of facilitators and barriers that affected maternal and newborn health services. The study findings warrant that there was a disparity in behavioral factors (awareness, beliefs, and behaviors) among community members, including pregnant women. This underscores the need to design health education programs and conduct social and behavioral change communication interventions to address individuals, families, and the broader community to enhance maternal and newborn health service utilization. On the other hand, the health sector should put into practice the available strategies, and health workers provide services with empathy, compassion, and respect.

## 1. Introduction

Ethiopia has made remarkable progress by achieving many national and global health indicators. The introduction of the Health Extension Program (HEP) has been one of the country’s innovative strategies used to accelerate the expansion of Primary Health coverage and serves as an effective mechanism for shifting health care resources from predominantly urban to rural areas, where the majority of the country’s population resides, to achieve the Millennium Development Goals. HEP was launched in 2003 to provide quality health promotion, preventive, and selected curative health services in an accessible and equitable manner to reach all segments of the population, with special attention to mothers and children [1,2].

The program is implemented at the health post level (i.e., a primary point of entry to the Ethiopian health system delivery tier in rural communities for providing health services for about 5000 populations). Two health extension workers (HEWs) are assigned at each health post and act as a primary point of entry to the health system for the rural community. They serve as the backbone of the Primary Health Care Units (PHCUs) acting as the most important frontiers for newborn and child survival in the country [3]. They recruited based on nationally agreed-upon criteria that included residence in the village, knowledge of the local language, graduation from 10th grade, and willingness to return to the village/kebele (i.e., Kebele is the lowest level unit of administration in the Ethiopian government which consists of 3000–5000 catchment populations), and serve the community. Two female trainees from the community are admitted to technical, vocational, and educational training institutions with short practical training in health centers; the training lasts a year. After graduation, they were assigned to their respective village to provide the HEP health services. The local governments pay salaries [1]. 

Their scope of practice ranges from the provision of preventive and promotive services to curative services. They spend 75 Percent of their time visiting families in their homes and perform outreach activities in the community. They spend the remaining 25 percent providing services at the health post, including immunizations and injectable contraceptives [1]. In addition, they provide curative services such as integrated common childhood illness management, including management of possible serious bacterial infections (i.e., sepsis management). They conduct activities under the close supervision of health workers from the health center [1,2,3,4]. 

Provision of proper maternal and child health care during pregnancy, delivery, and postpartum period has been one of the most important packages of continuum care to reduce maternal and neonatal morbidity and mortality [1,2,5]. At the grass-root level, health extension workers (HEWs) provide these services at the health post and home-to-home to reach all mothers and newborns [3,4,6] in support of the women’s health development army (WHDA). The WHDA is a network comprising women who have adopted better health behavior through completing the packages of HEP with five other women who have not yet completed so that they can influence the latter to practice a healthy lifestyle [1,3,4]. They support HEWs by notifying the presence of delivered women [7] and promoting and developing the health-seeking behavior of the community [2,3,4]. HEWs are expected to identify pregnant mothers, provide focused antenatal care (ANC) at health posts, promote institutional delivery, and provide immediate and essential newborn care, which includes cord care and application of Chlorhexidine, recognizing asphyxia, initial stimulation, and resuscitation of newborn babies. Management of pre-term and/or low-birth-weight neonates and promotion of Kangaroo Mother Care, management of Possible Serious Bacterial Infection when a referral is not possible, and early postnatal home visits, counseling and identification and care for sick neonates are also services to be provided by the HEWs [2,3,4,6]. 

Moreover, in collaboration with different partners, the Ethiopian Ministry of Health initiated a complex intervention called ‘Optimizing the Health Extension Program’, to improve the utilization of community-based child health services, such as community-based newborn care and integrated community case management of childhood illness services in Ethiopia. The program assumes that interventions conducted at the community level aiming to improve care-seeking would be most successful if they are linked to the health system and target both supply- and demand-side issues. Furthermore, it was hypothesized that: (1) engagement in demand creation activities and exposure to Information, Education and Communication or Behavioral Change Communication materials, would improve caregivers knowledge about child health danger signs; availability of child health services, and acceptability of the quality of primary care; (2) through capacity building (training, supportive supervision, performance, and clinical review meetings, and provision of job aids and tools and essential drugs for treatment of sick children) HEW would be knowledgeable, skilled, and motivated in managing sick children; and (3) through advocacy for the inclusion of child health indicators in annual district plans and budgets and the availability of ambulances for referral of severely ill children, districts would have improved ownership and accountability of child health programs. As a result of the synergistic effect of these three strategies, the overall utilization of child health services for sick children is expected to increase [8,9,10]. 

Existing evidence also indicates that the program brought a progressive and remarkable achievement, and strides improved access to health services and improvements in health outcomes [11,12,13]. However, despite the implementation of the program, newborn and maternal mortality rates are still unacceptably high. For example, the Ethiopia Mini Demographic and Health Survey conducted in 2019 indicated that the neonatal mortality rate was 30 deaths per 1000 live births [5]. However, this record was much higher than the global neonatal mortality rate in the same year (i.e., 19 deaths per 1000 live births). It was also slightly higher than the estimates in Sub-Saharan Africa and Southern Asia, where each reported 28 deaths per 1000 live births. Moreover, Ethiopia has been categorized as having a high concentration of neonatal deaths [14]. In addition, according to the 2016 Ethiopian demographic health survey, the country’s maternal mortality was 412 deaths per 100,000 live births [15]. This number is much higher than the global maternal mortality ratio (i.e., 211 per 100,000 live births, according to the 2017 estimate). However, it was slightly lower than the estimates recorded for sub-Saharan Africa, where two-thirds of maternal deaths occurred in the region with a ratio of 542 deaths per 100,000 live births. Ethiopian’s maternal mortality ratio was still much higher than the estimates recorded in Southern Asia, which had a ratio of 157 deaths per 100,000 live births [16]. 

Regarding maternal and newborn health services, utilization of four or more antenatal care, institutional delivery, and postnatal care check-ups in the first 2 days after birth was 43%, 48%, and 34%, respectively [5]. Pieces of evidence indicate that different barriers have contributed to the low utilization of maternal and newborn health services. However, there is limited evidence that explored the perceptions and experiences of HEWs on facilitators and barriers to these services. Therefore, this study explored the perception and experience of HEWs toward facilitators and barriers to maternal and newborn health care seeking and utilization. 

## 2. Materials and Methods

### 2.1. Study Setting and Period

The study was conducted in selected districts of Oromia, Amhara, and Southern Nation and Nationality People’s regional state regions of Ethiopia. Based on the 2007 Census conducted by the Central Statistical Agency of Ethiopia, these regions had an estimated total population of 26,993,933, 17,221,976, and 14,929,548, respectively. Regarding religion, the populations were Muslim (48%), Orthodox Christians (30%), and Protestant Christians (18%). The populations of Southern Nation and Nationality people’s regional state were Protestant (55.5%), Orthodox Christianity (52.86%), and Muslim (14.12%). The predominant religion of the Amhara for centuries has been Christianity, with the Ethiopian Orthodox Tewahedo Church (82.5%) playing a central role in the culture of the country followed by Muslims (17.2%) [17]. Data were collected on April 2021 from the three zones of these regions. The districts were selected from those settings where the Optimizing Health Extension Program was implemented [8,9]. 

Currently, Ethiopia follows a three-tier health care system: primary, secondary, and tertiary levels of care. At the grass-root level, there is a primary level of care (PHC) which includes primary hospitals (each serving 60,000–100,000 population), health centers (each serving 15,000–25,000 Population), and health posts (each serving 3000–5000 population). A primary hospital provides emergency, inpatient, and ambulatory services, and referral sites for health centers. It provides health care services for an average population of 100,000. In addition, it acts as a practical training center for nurses and other health care providers. Under the primary health care level, there is a primary health care unit (PHCU) that comprises one referral health center and five satellite health posts. Health posts are the lowest-level facilities in the healthcare system, and the point where PHC is administered and primary services are facilitated. Therefore, the HEP is a program designed to provide primary health care services in the nearby community. However, in an urban setting, the health care system is organized with a health center as the primary entry point (each serving about 40,000 people). The secondary health care system includes a general hospital (each serving 1–1.5 million people) that acts as a referral center for primary hospitals and also a training center for health officers, nurses, and emergency surgeons. A tertiary health care system includes a specialized hospital, a referral center for general hospitals (each serving 3.5–5 million people) [1,4,11,18,19].

### 2.2. Study Approach

A descriptive qualitative study was conducted to explore the perceptions and experiences of HEWs on the facilitators and barriers to maternal and newborn health service utilization at health posts. This approach was chosen because it is an important and appropriate design for research questions focusing on discovering who, what, and where of events or experiences that happened and gaining insights from informants regarding a poorly understood phenomenon [20]. It is also preferred because of its low time and resource consumption. A purposive sampling technique was used to recruit study participants. Data collection involved primary sources through semi-structured in-depth interviews and focus group discussions. The data analysis involved inductive thematic analyses. 

### 2.3. Study Participants and Sampling

A purposive sampling technique was used to recruit 60 HEWs from the three zones (Table 1). The HEWs were recruited based on certain criteria or considerations (i.e., inherently criterion/judgmental sampling technique) such as the number of the population they serve, level of education, work experience, diversity in distance from health centers, and performance. 

### 2.4. Data Collection Procedures

Data were collected through focus-group discussions. A semi-structured discussion guide containing 10 questions was developed to collect data. The data collection tool was initially developed in English and then translated into Afan Oromo, and Amharic languages. Finally, the English language expert back-translated it to English. The guiding questions were prepared primarily to address the following issues:

(A) Perceptions and experiences of HEWs toward community-related barriers affecting maternal and newborn health service utilization;
(B) Perceptions and experiences of HEWs toward community-related facilitators of maternal and newborn health service utilization;
(C) Perceptions and experiences of HEWs toward health facility-related barriers affecting maternal and newborn health services utilization;
(D) Perceptions and experiences of HEWs toward health facility-related facilitators to maternal and newborn health service utilization.
(E) Perceptions and experiences of HEWs toward infrastructure-related facilitators and barriers to maternal and newborn health service utilization.

Three individuals who had experience in qualitative research moderated the discussions; each in a different district town. In addition, three research assistants were involved in taking field notes and audio recordings. Twelve focus group discussions were conducted each comprising five participants. The group discussion lasted for a time ranging from 42 min to 67 min.

### 2.5. Data Analysis

Inductive thematic analysis, through which codes, sub-categories, categories, and themes were developed from the data, was employed to analyze the data. The analysis started by listening to the audio. Verbatim transcription was performed and field notes were simultaneously incorporated into the transcription. Then, the transcriptions were checked for completeness and consistency. After ensuring completeness and consistency, the transcriptions were translated from Amharic and Afan Oromo to English by another individual.

Reading and re-reading of the data were done to extract an important statement from the description and then coded line by line. First, the principal investigator and another assistant conducted line-by-line coding using ATLAS.Ti.7. Then, the given codes were checked for inter-coder consistency and a codebook manual was developed. The principal investigator then coded the data using the codebook manual to ensure code consistency and credibility. Potential major themes and sub-themes were developed by clustering sub-themes and codes, respectively to answer the research questions. The principal investigator repeated the coding system by refining the codebook manual, sub-themes, and themes. 

### 2.6. Trustworthiness

The trustworthiness of this study was ensured through credibility, dependability, transferability, and conformability principles [17,19,20,21,22,23,24]. The credibility of the study was ensured through peer debriefing, triangulation, and member checking. Research assistants, who had experience in a qualitative study, were involved in the data collection and analysis. Orientation was also provided to research assistants regarding the general purpose of the research and methodological procedures. The participants were also asked to summarize the major thematic findings of the discussion. Similarly, the modulator summarized the major points raised during the discussion, and the discussion was made on some unclear ideas at the end. Data were also obtained from health extension workers in different study settings.

To ensure transferability, thick descriptions were provided for the methodological procedures, interpretation of results, and contributions of research assistants. Therefore, the findings of this study can be applied to settings that have a health system and contexts similar to Ethiopia. Similarly, the dependability of the study is ensured through a thick description and audit trial. Moreover, the detailed chronology of methodological procures emerging themes; sub-themes or quotations were audited by qualitative research experts. The principal investigator was also not familiar with the study settings or participants. The findings of this study were presented in a way that the reader(s) can confirm through methodological procedures and findings. Furthermore, the findings are reported with supportive quotation(s) which opened a door for the reader to evaluate and build trust in the interpretations. In addition, the findings were audited and verified by qualitative research experts.

### 2.7. Ethical Considerations

Ethical approval was obtained from the Jimma University Institute of Health Institutional Review Board with the ethical code IHRPGD742/20. The principal investigator also took supports letters from the Ethiopian Public Health Institute and Regional health bureaus of the Oromia, Amhara, and Southern Nation and Nationality Peoples Region regions. The support letter helped the principal investigator to get acceptance of the respective zones and study participants. Written informed consent was obtained and participants were informed of the audio-recording and consent was obtained. Study participants were informed adequately about the purpose of the study, and the right to participate or withdraw at any time. To ensure their privacy and autonomy, codes were used instead of participant’s names during data collection and were informed that the study used the code in place of their name in connection with the study findings or their answers on discussions. Moreover, all COVID-19 preventive measures have been applied to prevent the infection. 

## 3. Results

### 3.1. Socio-Demographic Characteristics of Study Participants

Twelve focused group discussions were conducted and a total of sixty health extension workers participated in the study. Detailed characteristics of the participants are presented in Table 2. 

### 3.2. Facilitators and Barriers for Maternal and Newborn Health Service Utilization

This study found a wide range of facilitators and barriers to maternal and newborn health service utilization. Generally, for simplicity and to make it comprehensive for readers, the findings are presented in two major themes (i.e., Facilitators and barriers to maternal and newborn health care service utilization) and seven sub-themes (Table 3). However, there is no demarcation between themes and across sub-themes, and readers should understand this concept. The facilitators were also described first followed by barriers.

#### 3.2.1. Theme 1: Facilitators to Maternal and Newborn Health Care Service Utilization

##### Sub-Theme 1: Community-Related Facilitators for Maternal and Newborn Health Care Service Utilization

(i) Individual (pregnant woman) related facilitators

(a) Awareness

The study participants also mentioned that the majority of community members, including pregnant women, were aware of the importance and types of services provided during delivery, labor and delivery, and post-natal period. They also knew about the danger signs that happen during these times. In addition, it was mentioned that they were aware of the types of maternal and newborn health care services, including immunization services provided at the health post or home.

“*Having demand and awareness for the service provided is one of the facilitators. For example, a mother who knows the importance of immunization is utilizing more.*”

(27 years old, HEW)

(b) Behavior

Utilize the services: Study participants mentioned that pregnant women attend antenatal care, deliver at health facilities and receive postnatal care. They (pregnant women) attended pregnant women’s conferences, received vaccines, iron and were tested for sexually transmitted infections including human immunodeficiency virus (HIV) and syphilis. In addition, pregnant women were checked for hepatitis, diabetes mellitus, blood group, and Rh factor. They also tried to improve their nutritional status, maintain personal and newborn hygiene, stay at the maternity waiting room, and seek care from health facilities when any maternal and newborn danger signs had occurred. Furthermore, they seek care from health facilities for sick newborns. They saved money and prepared all the materials used during labor and delivery, including food items. 

Preparing for birth and birth-related complications: It was also mentioned that pregnant women save money as a means of birth and birth-related complications plan. It was mentioned that they save money through *Ekub* (i.e., rotating saving and credit association where a pot of funds is collected by a group of people who each contribute the same amount and then one person every meeting is selected at random to receive the pot.); *Edir* (i.e., a burial association established based on the mutual agreement of community members to collaborate whenever an adverse situation occurs in any member of the family.), Microfinance, or bank.

“*Yes. There are several saving options. For example, in our kebele, the health developmental armies collect money and save in the form of Ekub, and they give priority to the pregnant woman while raising it. She would also save money through Ekub with other members or Omo microfinance.*”

(21 years old, HEW)

Staying in the maternity waiting room: It was mentioned that, even if not all, pregnant women stayed at the maternity waiting room (i.e., residential facility, located near a health facility, especially health center, where women defined as “high risk” can await their delivery and be transferred to a nearby health facility (Health center or hospital) shortly before delivery, or earlier should complications arise. It is a strategy to “bridge the geographical gap” in obstetric care between rural areas, with poor access to equipped facilities, and urban areas where the services are available, and a low-cost way to bring women closer to needed obstetric care). They stayed there for days to months, and gave birth at the health facility.

“*These days, pregnant women go to the health center and stay at the maternity waiting room near term. All services are provided for free.*”

(20 years old, HEW)

(c) Experience

Pregnant women who had bad experiences with the outcome of their previous pregnancy utilized maternal and newborn health care from a health facility. For example, it was mentioned that women whose newborns died after home delivery gave birth at a health facility, or utilized maternal and newborn health care services more.

“*Even if it is not an exaggerated case, she [pregnant woman] would seek care from or deliver at the health facility if her newborn died previously after home delivery. Again, for the newborn, she might seek care if she had experience or know other women’s newborn died from not taking the vaccine.*”

(22 years old, HEW)

Similarly, it was reported that women who knew a woman who died from bleeding during home delivery utilized the service more.

“*For example, a pregnant woman who knows a woman who died from bleeding after home delivery might incline the supplementary fluid given at the health facility and demands to deliver there.*”

(37 years old, HEW)

On the other hand, HEWs mentioned that having bad experiences negatively affected maternal and newborn health service utilization. For example, it was mentioned that, in the study settings, culturally, most of the time pregnant women hide pregnancy until it becomes visible to people. However, if a pregnant woman tells others, including health workers or others, and starts to follow up early but faces abortion, she would not start antenatal care follow-up early for the next consecutive pregnancies. Similarly, it was mentioned that pregnant women whose newborns died from asphyxia during previous intuitional delivery, might not want to deliver at the health facility. 

For example, 26 years explained:

“*Previous obstetrics history is one of the reasons for a pregnant woman not to get delivery service from the health facility. They would not go to the health facility, for example, if her newborn was asphyxiated and died during her previous delivery.*”

Similarly, it was mentioned that there were pregnant women who did not give birth at the health facility if they did not face complications during their previous delivery at home. A 21 year old participant reported that:

“*In addition, mothers’ inability to deliver at a health facility is a lack of health problems encountered during the previous deliveries that happened at home. They perceive that since there was no problem encountered during the previous deliveries, no problem would encounter us during the current delivery.*”

(d) Satisfaction

The HEWs also reported that maternal and newborn health care service utilization was affected by the client’s satisfaction. It was mentioned that that community/clients’ who were satisfied with the service provided utilized them more than their counterparts. A 27-year-old HEW said:

“*The satisfaction obtained from the service provided for newborn and maternal health problems is one facilitator. For example, if the health facility provides appropriate or quality health services for mothers with the retained placenta or sick newborns and the clients are satisfied, the community develops a good attitude toward the services provided at the health facility.*”

(ii) Husband and family behavior

It was also reported that the husband and family support the pregnant women to regularly attend antenatal care visits, prepare for institutional delivery and seek care from the health facility. They collect and save money, help pregnant women to improve their nutritional status, maintain personal and newborn hygiene, provide social support, support her to take rest and avoid workload, motivate her to stay in the maternal waiting room; prepare traditional ambulances, facilitate/call for ambulances or other transportation services, and take her to the health facility during labor.

“*The husbands also play role in reducing their workload [of pregnant women], physical support, support to have different investigations, and supported in improving their dietary practice. The families also supported her in avoiding workload, improving her dietary practice by taking additional meals and enforcing to follow her health status.*”

(19 years old, HEW)

(iii) General community behavior

The study participants also mentioned that the community provides social support, prepares traditional ambulances, facilitates ambulance services, mobilizes resources, motivates pregnant women to stay in the maternity waiting room, and takes them to the health facility to receive services. 

It was mentioned that the community mobilized resources in cash or materials such as coffee, maize, cereals, and butter, as birth preparedness and complication readiness plan; to feed the pregnant women while staying in the maternity waiting room and after delivery. A HEW who was 19 years old reported that:

“*Facilitators are resources mobilized at the community level, coffee ceremony, health workers approach, availability and services provided in the maternity waiting room.*”

Similarly, other study participants had revealed that;

“*The other was that there was nothing that a pregnant woman took from home while she stayed in the maternity waiting room. This is because all necessary resources were mobilized from the farmers, and the health center prepared their [pregnant women] meals by employing servants. Therefore, they feel comfortable with the service and develop a positive attitude to give birth at a health facility.*”

(27 years old, HEW)

The study participants mentioned that the community members, including neighbors, supported pregnant women in conducting activities, helped them to take rest, serve children and prepare food while they stay in the maternity waiting room; prepare traditional ambulances (i.e., stretcher locally made from wood and robe or other materials) and take her to the health facility to give birth and facilitate ambulance or other means of transportation.

“*The family, as well as the community, support in taking her to the health facility, serving children at home and sharing the workload.*”

(21years old, HEW)

In addition, the community motivates pregnant women to regularly follow antenatal care, seek care and give birth at health facilities. Moreover, they provide social support, be with her at the health facility during labor and delivery which reassures her and feels the pregnant woman comfortable. A 26-year-old HEW had said that:

“*There are different facilitators among the community that makes mothers seek care for their own or newborn from the health facility…The community also motivates pregnant woman and tells her to analyze the benefits and risks of not going to the health facility during pregnancy, delivery, and post-delivery. Community members also go with her to the health facility during delivery. This is important to reassure and make her comfortable.*”

(26 years old, HEW)

##### Sub-Theme 2: Significant Others Related Facilitators for Maternal and Newborn Health Care Service Utilization

The study participants indicated different significant others who played a significant role in facilitating maternal and newborn health care service utilization. Majorly, they mentioned the role of traditional birth attendants, religious leaders, kebele chairmen, and women developmental army leaders.

(i) Traditional birth attendant behavior

According to the study participant’s experiences, previously, traditional birth attendants had encouraged pregnant women to give birth at home or assisted delivery at home. Currently, even if not all, traditional birth attendants motivate or encourage pregnant women to give birth at a health facility.

“*Previously, the traditional birth attendants said to the pregnant woman we provide you delivery service better than the health center. However, at the current time, we have involved them during pregnant women conferences and other discussions, and the problem is improved.*”

(19 years old, HEW)

(ii) Religious leader’s behavior

Religious leaders played a significant role in facilitating maternal health care service utilization. It was mentioned that they actively engaged in supporting and enforcing pregnant women to give birth at a health facility. In contrast, it was also mentioned that a few religious leaders still had no awareness or negative attitude toward institutional delivery, and challenged health care service utilization, especially institutional delivery.

“*Currently, religious leaders have improved their status and enforce pregnant women to give birth at health facilities. Some religious leaders said that what was the problem if a woman delivered at home? They also said that there is no need to go to a health center for a pregnant woman to get a delivery service. The major issue is awareness.*”

(28 years old, HEW)

(iii) Kebele chairman’s commitment

The study participants mentioned that the chairman of kebele worked together with the HEWs in mobilizing resources, facilitating pregnant woman identification, supporting/enforcing the community or pregnant women to seek care for maternal and newborn health care services from the health facility.

“*Our major role players and supporters are kebele leaders and health developmental armies. They facilitate resource mobilization, preparation of traditional ambulances and ambulance services.*”

(37 years old, HEW)

(iv) Women developmental armies’ behavior

The study participants also mentioned that the availability of a functional women development army facilitated maternal and newborn health care services. It was mentioned that the women’s developmental armies support the HEWs in identifying pregnant women, conducting PNC, identifying and referring sick newborns; identifying and referring women with danger signs; notifying birth that happened in the community/home; identifying and referring pregnant women for institutional delivery; creating awareness of the community and promoting service and developing health-seeking behavior of the community, especially pregnant women. For instance, 30 year old discussants reported that;

“*The women development armies supported us [HEWs] in supporting a pregnant woman goes to the health facility. They played a great role and called us [HEW] during labor. They also played a great role in preparing and supporting pregnant women for birth and delivery at the hospital.*”

##### Sub-Theme 3: Infrastructure Related Facilitators for Maternal and Newborn Health Care Service Utilization

(i) Telephone service

Access to the telephone service was mentioned as a facilitator of maternal and newborn health care. They reported that the community members call the HEWs any time or directly call for an ambulance service, especially for institutional delivery. Through this, they [community] access the maternal and newborn health care services provided at health facilities.

“*Previously, mothers give birth at home and die. However, currently, the community meets together, calls for an ambulance, and takes the pregnant woman to the health center during labor. This is the role of the community.*”

(20 years old, HEW)

(ii) Transport service 

Pregnant women use various means of transportation when access to ambulance services is difficult. It was mentioned that they use a Bajaj (three tire vehicles- those from near town), motor vehicles, public buses and cars from different sectors, or horses to go to the health facility. Those who live around the health facility would go on foot. The community, especially those who live far from the health facility or when access to transportation is difficult, also used traditional ambulances to take pregnant women to the health facility for delivery or other maternal and newborn health care services. All these types of transportation modalities have a significant contribution to the improvement of maternal and newborn health. For example, a 26 year old, HEW said:

“*Here, in the context of our community, since there is no transportation access at village areas, they use traditional ambulance, bed. They also use public buses like other people, which is not comfortable for her. The other, when she is around the asphalt area, she might get an ambulance service.*”

(iii) Power energy system

The availability of solar energy systems and other necessary materials facilitated service delivery. For example, it was mentioned that the solar energy system facilitated the provision of vaccines to the mother and newborn.

“*In the context of our Kebele or cluster health center, what I want to acknowledge is the district health office and different partners who supplied us solar energy system and television.*”

(32 years old, HEW)

##### Sub-Theme 4: Health Facility Related Facilitators of Maternal and Newborn Health Care Service Utilization

(i) Availability of health extension program

Study participants mentioned that the availability of HEWs at the health post facilitated maternal and newborn health services. It was mentioned that they provided antenatal care services (i.e., second and third ANC visits), referral services (i.e., first and fourth ANC visits, delivery, and other services), and provided postnatal care through home visits. They also create awareness about pregnancy, labor and delivery, and postnatal danger signs at the community level, promoting and providing health services. A 26 year old study participant reported:

“*In our Kebele, since the health extension program was started, there is no newborn death. For example, when newborns become sick, they can treat in the health post with Gentamycin and easily dispersible amoxicillin. Concerning mothers, similarly, it is good and if they become sick, they would be referred to the health center.*”

(ii) Health extension workers’ behavior to provide maternal and newborn health services

Early pregnancy identification: The HEWs also mentioned that they identify pregnant women as early as possible in support of the health developmental armies and Kebele leaders to provide maternal and newborn health services. They conduct this to provide antenatal care as early as possible or start it before 3rd month of pregnancy.

“*First, we tell the mother to come to the health center after she tells us about her pregnancy status or we identify the pregnancy through a home-to-home visit.*”

(29 years old, HEW)

Antenatal care services: Study participants mentioned that pregnant women received the second and third antenatal care from the health post, but were referred for the first and fourth visits. It was also mentioned that the HEWs provided Tetanus Toxoid [TT] vaccine at least twice, iron and folic acid tablets, and Mebendazole for de-worming for a pregnant woman, and checked her for blood pressure, nutritional status, and weight. A 32-year-old study participant said that:

“*After she has the first visit there [health center or hospital], I provide the second and third follow up, and again for the fourth follow up, we advise her to go to the health center or hospital to get the delivery service…We also teach her about danger signs that happen during pregnancy.*”

Delivery service: The study participants also mentioned that, generally, HEWs could not attend delivery at the health post. However, during precipitated labor or if a woman reached the second stage of labor and when the head of the newborn becomes visible, level IV trained HEWs could attend; given that if there is the glove and other clean materials. A 26-year-old study participant reported that:

“*Level IV HEWs can attend the delivery if the labor starts suddenly and the head of the newborn is significantly visible, and if there is a glove and other materials, but, an ambulance has not arrived early.*”

Postnatal care service: The HEWs also mentioned that they conduct postnatal care on the first, third, and seventh days through home visitation. It was indicated that they checked for maternal danger signs such as the presence of foul-smelling vaginal discharge, huge vaginal bleeding, headache, fever, loss of consciousness, lethargy, and convulsions. Similarly, they checked for newborn danger signs such as inability to breastfeed, loss of consciousness, fever, redness/pus around the umbilicus, skin rash, and convulsions, and they also provide awareness about these maternal and newborn danger signs to the mother and visit health facilities if any happened.

“*After delivery, the health extension workers can do four postnatal visits. Then, hand over to the cell leader for the next follow-up under close consultation with the health extension worker. On the first visit, the health extension workers teach her and can check the status of vaginal bleeding, about her hygiene, breastfeeding, umbilicus, and others. After the fourth visit, she gives to the cell leader for further follow-up.*”

(26 years old, HEW)

Referral service: HEWs refer clients for maternal and newborn health service utilization to the health center or hospital. For example, pregnant women are referred for the first and fourth antenatal care; Human immunodeficiency virus and syphilis test; diabetes mellitus, blood group, Rh factor, blood pressure, and hepatitis checkup. They also refer pregnant women to stay in the maternity waiting room and get delivery services. Similarly, a referral is done while a pregnant woman faces any danger signs.

“*During the postnatal period profuse vaginal discharge, high-grade fever, and lower leg and body swelling might happen. Therefore, during this time she should go to the hospital and set the service.*”

(30 years old, HEW)

“*We send her to the health center for the first and the fourth antenatal care visit and come to us for the second and third antenatal care. She receives services such as the provision of iron, vaccine, etc. both from the health post and health center.*”

(19 years old, HEW)

Immunization services: The HEWs mentioned that they provide vaccines both to the mother and newborn. For example, they provide TT vaccines at least twice for pregnant women. Similarly, they provided the BCG and Polio 0 vaccine for newborns. They also appoint the mother on the 45th day to start family planning and continue the rest immunization services for the newborn. A 21-year-old study participant reported that:

“…*Immediately after delivery, it would be facilitated for the newborn to get the BCG vaccine and Polio 0 at the health facility…We also provide advice to start immunizing the newborn on the 45th day*…”

Information: Study participants mentioned that HEWs provide awareness, counseling, and promotion services on maternal and newborn health services or conditions. For example, it was indicated that they provide awareness and counseling services on both maternal and newborn danger signs; the importance of antenatal care, institutional delivery, postnatal care, immunization; the importance of staying in the maternity waiting room, saving money, and preparing for birth, exclusive breastfeeding, optimal breastfeeding, nutrition, keeping personal and newborn hygiene and visiting health facilities if any health conditions occurred. They also promote services delivered at the community, health posts, health centers, and hospitals.

“*During the postnatal period, we also provide the service that was provided during the antenatal period. For example, we provide nutritional counseling and add one more meal than the ordinary. We advise the importance of exclusive breastfeeding. Second, if we recognize any maternal and newborn danger signs, we advise her to go to a nearby health facility immediately and receive appropriate services. The other is that we advise the mother to expose the newborn to sunlight.*”

(26 years old, HEW)

In addition, a 26-year-old study participant briefed that:

“*We advise her to expose the newborn to the sunlight and to breastfeed 10–12 times. Also, we advise her to completely breastfeed one breast before switching to the other. Other, we advise her not to start complementary feeding and feed only breast milk until six months.*”

Pregnant women conference: The study participants mentioned that there was a pregnant women conference conducted at the Kebele level. All pregnant women, health developmental army leaders, and women who gave birth participate in the conference and share different experiences. It was mentioned that through this conference, women receive different information and experience about pregnancy and pregnancy-related conditions that would benefit them to plan for institutional delivery, stay at maternity waiting home, prepare for birth, and be self-reassuring.

“*We conduct a pregnant women conference and make them share information and experience about their pregnancy, delivery and postnatal period to each other.*”

(20 years old, HEW)

Sick newborn treatment service: Study participants also mentioned that even if there is a shortage of medical supplies and equipment, there is a sick newborn treatment service at the health post.

“*There are many facilitators than the previous one…At all the health posts, ICCM (integrated community case management) service is provided free of cost for the newborn and children.*”

(31 years old, HEW)

(iii) Access to maternal and newborn services for free of charge

The study participants mentioned that, at the health post, all the services provided for both the mother and newborn are free. In addition, it was also mentioned that mothers freely get free service at the health center and in the maternity waiting room.

“*The services provided for free are preparing porridge and coffee ceremony to make the mothers feel at home and comfortable. The health facility also prepares foods preferred by pregnant women such as chicken, which motivates them to deliver at the health facility. The families would come to the maternal waiting room and different foods would be prepared for them, and through this they enjoy. This motivates them to deliver at the health facility.*”

(37 years old, HEW)

However, it was mentioned that there are health facilities i.e., health centers and hospitals that request payment for maternal and newborn health services, or they ordered to buy drugs from outside the health facility or private clinics.

“*The health center asks the pregnant woman to pay for the card during antenatal care visit. I have seen this issue and made health workers return birr. This stresses mothers.*”

(24 years old, HEW)

(iv) Incentives for giving birth in a health facility 

The participants also mentioned there different types of incentives given to a woman who gave birth at health centers. For example, baby towels or kits were provided, especially for those with economic constraints.

“*Even in the case when the delivered mother has an economical problem, baby kits would be provided for them. This motivates them to deliver at the health facility.*”

(32 years old, HEW)

(v) Accessibility of ambulance service 

Study participants have reported that accessibility of ambulance services facilitated maternal and newborn health services utilization, especially for institutional delivery and referral services. The community, health development armies, or HEWs called for ambulance service, and women get the services for free. In addition, they mentioned that there were occasions where women returned home after delivery through ambulance.

“*One of the facilitators access the ambulance service for free of cost and free service for drugs or service provided.*”

(28 years old, HEW)

(vi) Supervision and monitoring

One of the facilitators mentioned by the study participants was the availability of health centers and health post linkage. Through this system, health posts were regularly monitored or supervised by the health center and received feedback on the progress of the activities. However, they also mentioned that there are health posts far from the catchment health center that could not be supervised, monitored, or received feedback on regular basis.

“*There are a lot of facilitators. Availability of health posts at the Kebele level, availability of and the service provided by the health extension workers, health center and health post linkage… are all the facilitators.*”

(31 years old, HEW)

(vii) Availability of maternity waiting room and access to the provided services

The other commonly mentioned facilitators were the availability of maternity waiting rooms across health centers. They mentioned that there were all necessary materials there and pregnant women get services such as food, drink, etc., for free. Similarly, they were also allowed to stay in the maternity waiting room with a child or family members, and all service expenses were covered by the health center, and resources mobilized from the community. All food items were prepared as per the preference of pregnant women.

“*Previously, many mothers died from home delivery, prolonged labor, bleeding, and lack of access to transport. But, currently, the availability of ambulance services and maternity waiting room with blankets, televisions and other necessary materials facilitated mothers to seek care from health facility*”.

(25 years old, HEW)

#### 3.2.2. Theme 2: Barriers to Maternal and Newborn Health Care Services Utilization

##### Sub-Theme 1: Community-Related Barriers to Maternal and Newborn Health Services Utilization

(i) Distance

Study participants mentioned that there are kebeles located far from the health center to seek care. It was also indicated that there are health posts or health centers that were not constructed in the center of the community to serve all community members fairly.

“*After deliver, if the newborn develops a fever or becomes irritable, there is distance or difficulty of carrying it to seek care from the health facility.*”

(28 years old, HEW)

(ii) Topographic nature

The HEWs also mentioned that there were areas with topographic difficulties and were not comfortable for pregnant women to go to a health facility. In addition, it is very difficult to access any means of transportation, including access to ambulance services, and the community faces challenges in taking pregnant women.

“…*Also, there is a topographical difficulty and distance from a health facility. It is very difficult even for us [normal people or non-pregnant women], not only for a pregnant woman. It is difficult even to use traditional ambulance because of its sloppy and hill.*”

(37 years old, HEW)

(iii) Workload

Study participants reported that pregnant women were overloaded with both outdoor and indoor activities. It was mentioned that since there are husbands and family members who do not support them to conduct activities, they do not want to stay in the maternity waiting room; they travel far for antenatal care, institutional delivery, and postnatal care. A 31 year old respondent explained:

“The husband might not support her in conducting different activities. Some women are overloaded with different activities at home or outdoors. Therefore, we conduct discussions with the husbands, families [mother and father], mother-in-law, religious leaders, and influential persons, and identify the barriers. We also create awareness about maternal and newborn health services.”

(iv) Economical constraint

The HEWs mentioned that there were pregnant women who could not utilize the service due to a lack of money for transportation, birth preparation, or other purposes. They overcome this problem by borrowing from their neighbors.

“*In the context of my kebele, there are also those who know their date of delivery but do not have anything at home to prepare for birth. Also, the husband goes to the neighbor during labor to find something important during delivery.”*

(31 years old, HEW)

(v) Women’s power of decision making

The participants mentioned that there was a husband or family who had wrong perceptions toward maternal and newborn health services delivery, especially for institutional delivery. They do not support or allow pregnant women to seek care for both maternal and newborn health conditions. For example, it was mentioned that there was a husband or family/mother-in-law who said to a pregnant woman that all the family members were born at home and no need to go to a health facility for giving birth. Moreover, mothers-in-law resist pregnant women not attending antenatal care or giving birth at the health facility and challenge the HEWs during family discussions.

“*There are mothers-in-law who complain that they gave birth of 10 or 13 children at home and resist the pregnant woman to deliver at home.*”

(28 years old, HEW)

(vi) Religious and socio-cultural beliefs and practices 

The HEWs mentioned that religious and cultural beliefs and practices affected the services delivery. There were settings where pregnant women could not expose their naked bodies to others, especially Muslim religious followers. This affected antenatal care, institutional delivery, or postnatal care service utilization.

“*According to our community, it is forbidden to see a female’s bare body other than her husband or brother. Therefore, they prefer to deliver at home.*”

(32 years old, HEW)

There are also traditional practices that affected service deliveries. For example, it was mentioned that, in some settings, there was a celebration conducted by the family of women who gave birth on the second birthday. They slew hen and bruit beneath the bed of the delivered woman. It was mentioned that this celebration is conducted only for those women who delivered at home, and they perceive that it would protect both the mother and newborn from evil spirits. A 32 year old Study participant said that:

“*There are factors related to this issue that affects maternal and newborn services delivery. For example, there was a pregnant woman who challenged us to give birth at a health facility and delivered at home. On the next day after delivery, when I went to give postnatal care, they bruited slain hen under her bed. This is a traditional practice conducted within the community for women delivered at home. Therefore, we should continue to provide the service and change the practice through the process. I know that there was a woman who wanted to slay hen at the health center after giving birth. During this time, if we do not allow her to do this, they would not come to the health center.*”

Similarly, there were traditional practices conducted among Muslim religious followers. It was mentioned that there is a celebration so-called ‘porridge celebration’ conducted for pregnant women on the 7th and 9th months of pregnancy. They celebrate it at home by cooking and eating porridge. It was conducted to bless a pregnant woman to face good birth outcomes/deliver safely. Therefore, because of this; pregnant women do not want to stay in the maternity waiting room, and perceive that a woman might have died if it was not celebrated or blessed. A 32 year old participant reported that:

“*In the context of our Kebele, the Muslim community does not allow pregnant women to stay in the maternity waiting room or deliver at health facilities. They complained that there is a porridge ceremony on the 7th and 9th month and do not go to a health facility. Due to this, they perceive that the pregnant woman would die if she goes and delivered at a health facility.*”

On the other hand, the community did not allow pregnant women to give birth at health facilities due to different perceptions or misconceptions. For example, the community misconceives the white coat on the body of the newborn as a dirty substance. Since it is not recommended to bathe the newborn before 24 h and the mother returns home before this hour, they perceive that she brings the newborn with a dirty substance.

“*There are community’s traditional practices that affected intuitional delivery. For example, if a newborn is born at a health facility, it is not bathed within 24 h unlike that of newborns born at home. So, the community perceives that it comes home with the dirty body.*”

(22 years old, HEW)

Similarly, it was mentioned that the community relates it to evil spirits while pregnant women face health problems, and take them to religious leaders rather than a health facility. For example, it was mentioned that if a pregnant woman experiences bleeding during pregnancy, they perceive that she contracted an evil spirit and take her to the religious leaders.

“*The community also perceives as an evil spirit contracted the women if she faces bleeding (APH) during pregnancy and takes to the religious leaders. Therefore, since the religious leaders and other community leaders are accepted by the community, it affected service utilization.*”

(19 years old, HEW)

There were food taboo practices that were against nutritional counseling services/recommendations for pregnant women. It was mentioned that HEWs counsel pregnant women to eat more meals during pregnancy and postnatal periods. However, it was indicated that there were food items not allowed for a pregnant woman to eat. For example, in a certain study setting, pregnant women were not allowed to eat sugarcane (i.e., perceived to lead prolonged labor), take hot drinks (i.e., perceived to result in newborn baldness), porridge (i.e., perceived grafted on the newborn body), and pimento (i.e., perceived burn the newborn).

“*There are mothers not allowed to eat foods like sugar cane, spice, cabbage, etc. For example, there is a belief that if women eat sugarcane during pregnancy, she [the pregnant women] would face prolonged labor; if she[the pregnant women] drinks hot drinks, the newborns become bald; if she [the pregnant women] eats pimento, it would burn, if she [the pregnant women] eats porridge, it would be stickled on the newborn’s body. Therefore, we have to remove this belief from her mind and make her eat all these foods.*”

(19 years old, HEW)

(vii) Unpleasant rumor 

On the other hand, it was also mentioned that communities’ health services utilization for maternal and newborn health was affected because of unpleasant rumors from poor quality of health services or health workers’ approach. For example, it was mentioned that a woman contaminates others not to utilize health care service from the health facility if mistreated at the maternity waiting room or during delivery.

“*Rumors might be disseminated within the community. For example, if a woman died at a health center or hospital, others might perceive that she died due to going to a health facility or from poor health workers handling. Due to this, they might not go to the health facilities.*”

(21 years old, HEW)

##### Sub-Theme 2: Health Facility-Related Barriers to Maternal and Newborn Care Service Utilization

(i) Health care provider’s behavior

Empathy, compassion, or respect: Health worker’s discipline, compassion, or approach was one issue majorly raised by the study participants as a barrier. It was mentioned that there were health workers who disrespect/insult pregnant women or lacked compassion while providing services to clients. They postpone the follow-up date from hours to weeks; delay in providing the service, including referral service; providing the service unfairly; disrespect/insulting the client, and not keeping the hygiene of delivery beds or room. They emphasized that those clients disrespected by the health workers would contaminate others not to seek care from that health facility; which affected the service delivery. A 24 year old study participant reported that:

“*Even if it is not all, there are some health workers that disrespect the pregnant women. They also tell her to return after she comes from far. Again, they tell her as the health worker goes site, and this is an obstacle for a pregnant woman to seek care. Due to this, she might become angry or negligent.*”)

The health care providers asked for the cleanliness of pregnant women:

“*They would say you do not bathe your body or hair or smell foul. Why you do not come changing your cloth or shoes? Due to this reason, they fear coming to the health facility. There is anger among the health workers.*”

(29 years old, HEW)

Fairness or equality: On the other hand, it was mentioned that the health workers provide the service inequitably. They gave priority to those who wear well or were perceived as rich.

“*The barrier from the health center or hospital side is that the health workers do not provide the service equitably. This means that they provide service judging based on the wearing style of a pregnant woman…They provide drugs or other services for those who wear clean clothes but order others to buy from outside. Is that the drug or other services are given for free are only for those who wear well? Not for those who come from the village [rural]? What our community has are mouth and hand, and if they buy drugs, even they might have nothing used for transport to return to home. This needs special attention and these are all the barriers.*”

(29 years old, HEW)

Absenteeism: The participants also mentioned that they refer clients to the health center or hospital for antenatal care, delivery, or postnatal care. However, the health workers assigned at this specific clinic might not be present at the right time. Therefore, clients cannot get the service or delay in getting the appropriate service. Due to such issues, it was mentioned that there were clients who did not go to the health center or hospital.

“*Again, they tell her [pregnant woman] that the health worker goes outreach, and this is an obstacle for a pregnant woman to seek care. Due to this, she might become angry or negligent to utilize the service.*”

(27 years old, HEW)

(ii) Lack of adequate medical supplies and equipment’s at the health post

The study participants mentioned that there are level IV trained HEWs at the health posts who can attend the delivery. However, it was mentioned that there was no quality or adequate medical supplies and equipment at the health post to provide a safe and clean delivery service.

“*At the health post, there is lack of quality or clean equipment. Pregnant woman should have to deliver by clean materials.*”

(26 years old, HEW)

Similarly, it was mentioned that there was a shortage of drugs or medical supplies to treat sick newborns and children as per the integrated community case management and community-based newborn care guideline. For example, a 28 year old HEW said that if a mother comes to seek care for a sick newborn from a health post, there is no drug. She, in turn, contaminates her neighbors not to go and seek care from health post.

“*While we implement ICCM (integrated community case management), there is a shortage of drugs…For the children above 6th month, for example, Plump nut and other drugs are provided. But, the supplies or drugs might not be available.*”

(28 years old, HEW)

(iii) Placement and quality of health post

Study participants also mentioned that there were health posts constructed not centering the community. Due to this case, only those clients who live nearby the health post utilize the service. On the other hand, there were health posts that had poor quality or were poorly maintained, and unable to attract the community to seek care for maternal and newborn health services from there.

“*The health post is not constructed in the middle of the community. The second barrier is that there is no difference between constructed health posts with the house of the community except the materials available within it. It has nothing that we put the materials. This is because there are rats, which destroys the materials available at the health post.*”

(19 years old, HEW)

##### Sub-Theme 3: Infrastructure Related Barriers to Maternal and Newborn Health Care Service Utilization

(i) Lack or poor quality of road

Study participants mentioned that certain kebeles had a lack or poor quality of roads, and challenged utilization of maternal and newborn health services. Pregnant women were facing the difficulty of accessing any means of transportation, including ambulance services, especially, during the summer period; they face difficulty in crossing rivers to utilize the service. A 35-year-old study participant has reported that:

“*The barriers to seeking care are a distance from the health facility, inability to cross a river during summery from flooding…Due to this, there is maternal death or stillbirth.*”

(ii) Lack of water supply at the health post

Study participants mentioned that there was no water supply at health posts to provide clean and quality care both for the mother and newborns.

“*The other barrier is lack of water at the health post.*”

(28 years old, HEW)

(iii) Inadequate ambulance services

Study participants mentioned that there was a shortage of ambulances. Thus, pregnant women did not get adequate ambulance services. They mentioned that while they call for an ambulance the district health office complains as there was no tire, benzene, or went for other referral or another purpose. It was mentioned that clients were suffering, especially those from very far from the health center, during the night time; given that there is no transportation access at night.

“*The available ambulance if not enough or it is a big challenge. Sometimes, they say that there is no benzene, tire or the ambulance goes for referral purpose.*”

(29 years old, HEW)

## 4. Discussions

The study explored a wide range of facilitators and barriers of MCH services from both the community and health facility side. There is no clear demarcation in between the findings as a theme or across sub-themes. Moreover, a discussion was made according to the interrelatedness of concepts across themes or sub-themes. 

Awareness was one of the findings explored in this study. From the study, it was understood that there were disparities among the community members, including pregnant women in awareness related to maternal and newborn danger signs, availability, and importance of maternal and newborn health services at the health post. This underscores the need to design and conduct health education programs/campaigns to be undergone at different opportunities to make all community members have enough knowledge and minimize the knowledge gap. Similarly, existing evidence indicated that knowledge/awareness is a significant determinant for maternal and newborn health services [22,25,26,27,28,29,30,31,32,33,34,35,36]. 

The study also found that pregnant woman utilizes maternal and newborn health services, and supported by husband/family and community to get the services. This might imply that the program has been accepted by the community members. However, it was explored that there was a disparity among pregnant women in utilizing the services, and in getting support from the husband/family and community. Therefore, this underscore, there is a need to design and conduct a community-based social and behavioral change communication intervention to address the target population of the community and actively engage or participate the community members to enhance the utilization of the services. Similarly, different studies support this finding that gaining support or motivation from family, husband, and community was a key positive or negative determinant for maternal and newborn health services utilization [26,27,28,33,36,37,38]. 

The study also explored that the behavior of significant others such as traditional birth attendants affected either positively or negatively the maternal and newborn health services, especially for institutional delivery. It was indicated that there was a disparity among traditional birth attendants in supporting pregnant women to get skilled birth attendants. This implies motivation is required for those traditional birth attendants who support positively, and there is a need to design and conduct social and behavioral change communications to address those who negatively influence maternal and newborn health service utilization. Similarly, existing evidence indicated that traditional birth attendants were a key determinant for maternal and newborn health services [25,28,29,35,36]. 

Similarly, there was a disparity among religious leaders, significant others, to support, motivate or enforce pregnant women to give birth at the health facility. This implies that religious leaders positively or negatively affected the utilization of maternal and newborn health services. This underscores there is a need to design and conduct a social and behavioral change communications intervention to address these segments of the population and actively engage to enhance maternal and newborn service utilization. Similarly, different studies indicated that the role of religious leaders was a key determinant to maternal and newborn health service [22,26,28,33]. 

Pregnant woman’s experience from the previous pregnancy outcome also affected their utilization of maternal and newborn health services. For example, it was found that those women who experienced abortion or bad birth outcomes from previous pregnancy after early initiation of antenatal care or during institutional delivery, respectively, did not utilize the service and vice versa. In contrast, those women who had an experience of knowing women contracted bad birth outcomes, including death, from home delivery or not seeking care from the health facility utilize maternal and or newborn health services and vice versa. This implies that one’s experience from the outcome of the previous pregnancy matters utilization for maternal and newborn health services. However, existing evidence indicated that all pregnancy is at risk and it is difficult to judge who should face the health problems [2,6]. Therefore, this implies a need to design and conduct behavioral change communication intervention programs and change this perception and behavior. 

Infrastructure was also explored both as a facilitator and barrier to maternal and newborn health service. The study found that availability/access to telephone service and lack or poor road quality was a facilitator and barrier, respectively. It was found that availability of telephone service has significant importance to utilize the service through facilitation of access to ambulance service and referral service. However, it was mentioned that the service delivery was affected by distance, topographical difficulties, or road problems. This nature of the study settings affected access to any means of transportation, including ambulance, motor, etc. to utilize the service although the study found availability access to different means of transportation as a key facilitator. It was indicated that the community used a traditional ambulance to take pregnant women and utilize health services. Similarly, these findings are in line with the findings of different studies which indicated that distance, topographic, road, lack of transportation, etc. affected maternal and newborn health services [22,25,26,28,33,34,35,36]. 

Different religious and socio-cultural beliefs and practices affected maternal and newborn health service utilization. There was a disparity in religious and socio-cultural beliefs and practices in different study settings. Similarly, the disparity was also there related to religious and socio-cultural practice during prenatal, delivery, and postnatal periods. This implies that there is a need to actively engage the religious leaders, opinion leaders, community leaders, and other religiously or culturally respected/influential persons in the health care system to play a key role as a change agent. In addition, since there is a disparity among the community members, including the pregnant women to utilize the service, there is a need to use those who utilized the service as a role model, change agent, or a ‘positive deviant’ to promote the service delivery. Therefore, this also needs a designing and conducting of social and behavioral change communications interventions by actively engaging such segment of the community to enhance service utilization. Similarly, existing evidence indicated that religious and socio-cultural beliefs and practices affected health service delivery. However, it was context or culture-based or religious and socio-cultural beliefs and practices vary across different settings and cultures [22,25,26,28,33,34,39]. 

The study also explored that the women’s developmental armies’ behavior, significant other, facilitated maternal and newborn health services in the study settings. Similarly, a conducted in Ethiopia showed the women’s developmental army has been conducted different maternal and newborn-related activities, including identification of sick newborns, provision of postnatal care, identification of pregnant women, promotion of health services, etc., at different settings [2]. In contrast, a study conducted in the Debre Libanos district indicated that there were no functional health developmental armies that support the health extension workers in identifying pregnancy, promotion, and provision of service, and facilitate linkage between client and health facility [22]. This difference might be due to geographical differences. 

It was found that the availability of HEWs to provide maternal and newborn service, including sick newborn treatment as per the health extension program guideline. It was found that these services are provided for free [1,2,6]. 

The study also found that maternity waiting rooms at the health center at which pregnant women stay for a certain period of time (i.e., near term) to get skilled delivery. The community mobilizes resources used to serve pregnant women and their families at maternity waiting rooms for free. Similarly, different study findings support this finding that it was one of the key determinants for maternal and newborn service, especially institutional delivery [26,36]. However, there was a disparity among pregnant women to stay in the maternity waiting room and get delivery service from a health facility. This underscores that there is a need to design social and behavioral change communication interventions to create a favorable environment for pregnant women to stay in the maternity waiting room and get skilled delivery. This is because it is through which barriers like distance, topographic difficulty; problem-related to the road, and transportation access would be solved [26,36,40,41,42,43,44,45]. 

It was explored that there was a lack of adequate medical supplies and equipment that affected maternal and newborn health care utilization, especially to attend the delivery, and also provide service for sick newborns at the health posts. This implies that there is an interruption of the services provided due to the supply chain system. Therefore, there is a need for strong monitoring and supervision of the supply chain system, and refill the required or necessary materials needed at the health post. Similar problems were reported in different studies [22,28,33,35].

The study also explored that there was disparity or irregularity of supportive supervision and monitoring provided to the HEWs from the health centers. However, it is expected that the HEWs should be supervised on a weekly basis by health workers from the health center [2]. Similarly, a study conducted at Debre Libanos district, Ethiopia, supports this finding [22]. Therefore, this underscores the need to put into practice the existing principles and strategies endorsed by the Ministry of Health. 

Infrastructure was also mentioned as a key determinant for maternal and newborn health service utilization. For example, it was indicated that the availability of solar energy systems facilitated immunization services. In contrast, service provision was affected by a lack of access to the water supply. Therefore, there is a need to fulfill all the necessary infrastructures needed at the health post level to provide quality service for both the mother and newborn. Similarly, other study findings support this finding that HEWs complained about a shortage of equipment, electric power, and water supplies [46].

Payment request was the other explored barrier in this study. It was indicated that clients were requested to pay for maternal and newborn health services they obtained, especially at the hospitals. In contrast, according to the Ethiopian government health policy, pregnant women are not requested for health services obtained from the public health facilities for pregnancy and pregnancy-related conditions. Therefore, this indicates that the health workers did not stick to the policy endorsed by the government; which warrants a need to put into practice the initiatives and provide the service to the maternal and newborn to reduce morbidity and mortality. Similarly, there was a study that supports this finding in that hospitals demanding payment for delivery services [25]. 

Health workers’ behavior was found as both facilitator and barrier. There was a disparity of compassion, empathy, and respect among health workers towards clients seeking care. Clients who obtained compassion, empathy, or respect from health workers utilize the maternal and newborn service and vice versa. The study also found that this health worker’s behavior created rumors within the community that affected the service utilization. This implies that the health workers should not understand that it is their responsibility to keep clients’ dignity and respect, and the client’s right to be treated with care, compassion, and respect. Therefore, this underscores that there is a need to provide a patient-centered service for clients in need of maternal and newborn health care services. Similarly, this finding is supported by other study findings which indicated that health service utilization was determined by health worker’s approach, compassion, and empathy [47,48,49].

Women’s decision-making power was also explored as a barrier to utilize maternal and newborn services. The study found that most of the time the decision to uptake the services was determined by the husband and or mothers-in-law. These needs designing a health education program and implement social and behavioral change communications to address such segment of the community to create a favorable environment that supports or motivates women to utilize health care services during pregnancy, delivery, and postnatal period. Similarly, this finding is supported by different study findings [25,26,27,28,29,30,33,35,36,38]. 

## 5. Conclusions

The study explored a wide range of facilitators and barriers to the maternal and newborn health service. There was no demarcation between the facilitators and barriers. The service utilization was facilitated from behavioral factors (awareness, beliefs, and behavior) held at the individual (pregnant women), family, community, and significant others. This implies that there is a need to design health education programs and conduct social and behavioral change communication interventions to address individuals, families, and the broader community to enhance maternal and newborn health service utilization. On the other hand, service delivery was affected due to lack of adequate supply chain system, health workers discipline, or supervision and monitoring. Thus, the health sectors should have to put into practice the available strategies and fulfill all the required infrastructure and logistics. Moreover, health workers also should have to provide quality service with empathy, compassion, and respect.

## Figures and Tables

**Table 1:** Distribution of health extension workers (HEWs) who participated in the study to explore barriers and facilitators to maternal and newborn health service utilization, Ethiopia, 2020.

Region	Zones	Districts	Number of HEWs Recruited	Number of Kebeles
Oromia	West Hararge	Chiro	20	20
Amhara	North Shoa	Tasma Ber	20	20
SNNPR	Dawro	Loma	20	20
Total	3	3	60	20

**Table 2:** Socio-demographic characteristics of health extension workers participated, Ethiopia, 2021 (*n* = 60).

Characteristics	Category	Number (*n*)	Percent (%)
Age	≤20 years	5	8.3
	21–30 years	25	41.7
	31–40 years	30	50
Level of training	Diploma (level III)	28	46.7
	Level IV	32	53.3
Work experience	<5 years	13	21.7
	5–10 years	22	36.7
	11–15 years	25	41.7

**Table 3:** Summary of major themes, sub-themes, and concepts generated from a study conducted to explore facilitators and barriers for maternal and newborn health care service utilization in Ethiopia, 2021 (*n* = 60).

Major Themes	Sub-Themes	Concepts
Facilitators to maternal and newborn health services	Community-related facilitators for maternal and newborn health care service utilization	
	Individual (pregnant women) related facilitators	Awareness
		Behavior (Utilize the services, prepare for birth and birth-related complications, and stay at maternity waiting room)
		Experience
		Satisfaction
	Husband or family behavior	Resource mobilization
		Social support
		Motivational support
	General community behavior	Resource mobilization
		Social support
		Motivational support
	Significant others related facilitators for maternal and newborn health care services utilization	
	Traditional birth attendant behavior	
	Kebele chairman’s commitment	
	Religious leaders involvement/behavior	
	Women developmental army behavior	
	Infrastructure related facilitators for maternal and newborn health services utilization	
	Telephone service	
	Transportation service	
	Power energy system	Solar energy system
	Health facility related facilitators to maternal and newborn health care services utilization	
	Availability of health extension program	
	Access to and health extension workers behavior to provide maternal and newborn service	Antenatal care
		Delivery service
		Postnatal care
		Referral service
		Immunization service
		Information
		Pregnancy identification
		Pregnant women conference
		Sick newborn treatment
Facilitators to maternal and newborn health services	Access maternal and newborn health services for free	
	Incentives	
	Access to ambulance service	
	Supervision and monitoring	
	Access to maternity waiting room and service	
Barriers to maternal and newborn health care service utilization	Community-related barriers to maternal and newborn health care services utilization	
	Distance	
	Topographic nature	
	Workload	
	Economic constraint	
	Woman’s power in decision making	
	Religious and socio-cultural beliefs and experience	
	Unpleasant rumor	
	Health facility-related barriers to maternal and newborn health care services utilization	
	Health workers behavior	Empathy
		Compassion
		Respect
		Postponement
		Fairness and equality
		Absenteeism
	Lack of adequate medical supply and equipment	
	Lack of adequate ambulance service	
	Placement and quality of health post	
	Payment request	
	Infrastructure related barriers to maternal and newborn health care services utilization	
	Lack or poor quality of road	
	Lack of water supply at the health post	
	Inadequate ambulance	

## Data Availability

All study data were reported in the tables within the manuscript.
